# Scrotal Lymphedema

**Published:** 2011-11-04

**Authors:** Craig Pastor, Mark S. Granick

**Affiliations:** Division of Plastic Surgery, University of Medicine and Dentistry of New Jersey, Newark, NJ

## DESCRIPTION

A 39-year-old man presents with a several-year history of an enlarged scrotum that developed after a bout of epididymitis.

## QUESTIONS

1. **Describe the pathophysiology of scrotal lymphedema.**
2. **What are the common causes of scrotal elephantiasis?**
3. **What treatment options are available for this disorder?**

## DISCUSSION

Scrotal elephantiasis, or massive scrotal lymphedema, is a disease that is caused by obstruction, aplasia, or hypoplasia of the lymphatic vessels draining the scrotum. The scrotal skin is thickened and may exhibit ulcerations in severe cases. It can be either congenital or acquired in nature, with the most common acquired etiology being infection. The most common infections leading to scrotal elephantiasis are lymphogranuloma venereum or filarial infestation with Wuchereria bancrofti. The rare occurrence of these infections in Western nations makes scrotal elephantiasis an uncommon disease outside of Africa and Asia. Other causes of this disease include chronic inflammation, neoplasm, irradiation, and lymph node dissection.

Treatment of this condition is guided by the etiology. Response often depends on whether the lymphatic derangement can be reversed. In cases where the lymphedema is caused by fluid overload or congestive heart failure, diuretics can be of benefit. Mild and acute cases due to sarcoidosis may benefit from steroids. Antibiotics may be all that is necessary in cases of acute infection. When the lymphedema is chronic, with resultant skin and subcutaneous fibrosis, more aggressive therapy is warranted. There are several surgical options. In most cases requiring surgery, the skin is involved and needs to be removed. The testicular subcutaneous tissue is indurated and full of lymphatic fluid and similarly needs to be removed. The testicles and spermatic cord are generally preserved and unaffected by the lymphedema. However, in some cases, the penile skin can be chronically avulsed off the penile shaft by the weight of the affected scrotum, as in our case. The penile shaft should be split-thickness skin grafted when it has been denuded in this fashion. The testicles can be implanted in the thighs or lower abdomen unless there is sufficient residual tissue to reconstruct a scrotal sac. If the testicles are replaced into a neo-sac, then they must be pexed to prevent torsion.

Our patient is a 39-year-old man who presented to the plastic surgery office with a several-year history of an enlarged scrotum that extended to his knees. The patient's penis was completely obscured by the scrotal tissue and his urinary stream emerged from a tunnel of avulsed penile shaft skin embedded in his scrotum. The patient denied travel to areas endemic with *Chlamydia trachomatis* or *Wuchereria bancrofti* but reported that following a case of epididymitis, his scrotum began to enlarge progressively. Because of the chronic nature of the patient's disease and the irreversible changes to his skin and subcutaneous tissue, he would not have benefited from conservative management. The patient underwent excision of scrotal skin and subcutaneous tissue, orchiopexy, skin graft to his penis shaft, and reconstruction of his scrotum with perineal skin that had been spared from the disease process.

## Figures and Tables

**Figure:**
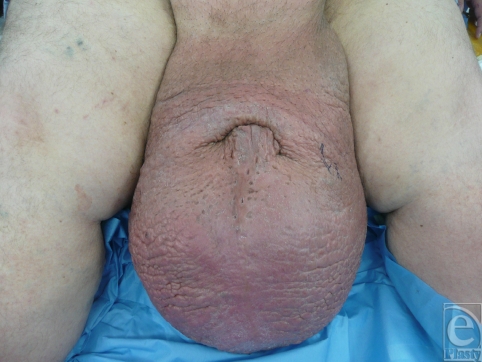


**Figure:**
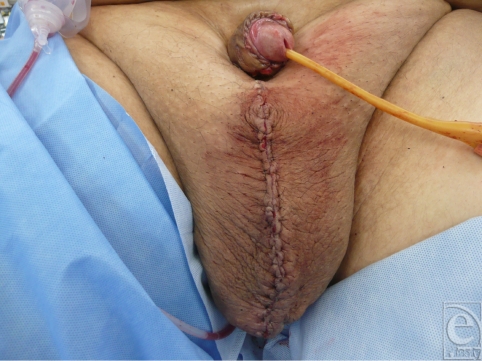


## References

[1] McDougal SW (2003). Lymphedema of external genitalia. J Urol.

[2] Hornberger BJ, Elmore JM, Roehrborn CG (2005). Idiopathic scrotal elephantiasis. Urology.

[3] Denzinger S, Watzlawek E, Burger M, Wieland WF, Otto W (2007). Giant scrotal elephantiasis of inflammatory etiology: a case report. J Med Case Reports.

[4] Zacharakis E, Dudderidge T, Zacharakis E, Ioannidis E (2008). Surgical repair of idiopathic scrotal elephantiasis. South Med J.

